# Evaluating Health Disparities in Radiology Practices in New Jersey: Exploring Radiologist Geographical Distribution

**DOI:** 10.7759/cureus.43474

**Published:** 2023-08-14

**Authors:** William J Lee, Yash Shah, Albert Ku, Nidhi Patel, Magdalena Salvador

**Affiliations:** 1 Radiology, Rutgers University New Jersey Medical School, Newark, USA; 2 Radiology, Drexel University College of Medicine, Philadelphia, USA

**Keywords:** public health care, underserved populations, radiology, health care disparity, physician shortage

## Abstract

Objective: This study aimed to determine if a disproportionate number of radiologists practice in high-income versus low-income counties in New Jersey (NJ), identify which vulnerable populations are most in need of more radiologists, and discuss how these relative differences can ultimately influence health outcomes.

Methods: The NJ Health Care Profile, a database overseen and maintained by the Division of Consumer Affairs, was queried for all actively practicing radiologists within the state of NJ. These results were grouped into diagnostic and interventional radiologists followed by further stratification of physicians based on the counties where they currently practice. The median household income and population size of each county for 2021 were obtained from the US Census database. The ratio of the population size of each county over the number of radiologists in that county was used as a surrogate marker for disparities in patient care within the state and was compared between counties grouped by levels of income.

Results: Of the 1,186 board-certified radiologists actively practicing within the state of NJ, 86% are solely diagnostic radiologists and 14% are interventional radiologists. About 44% of radiologists practice within counties that are within the top one-third of median household income in NJ, 25% practice within counties in the middle one-third, and 31% practice within counties in the bottom one-third.

Conclusions: There is a disproportionate number of radiologists practicing in high-income counties as opposed to lower-income counties. A contradiction to this trend was noted in three low-income counties: Essex, Camden, and Atlantic County, all of which exhibited low numbers of individuals per radiologist that rivaled those of higher-income counties. This finding is a concrete measure of successful radiologist recruitment efforts within these counties during the past few years to combat the increased prevalence of disease and associated complications that historically marginalized communities tend to disproportionately exhibit. Other low-income counties should look to what Essex, Camden, and Atlantic County have done to increase radiologist recruitment to levels that rival those of high-income areas.

## Introduction

The growing shortage of physicians in the United States and the implications this trend has on overall patient health has been well documented. Patients who are residents of areas deemed to have a shortage of primary care physicians, which are typically low-income or rural areas, have already been shown to have increased morbidity and mortality due to heart disease, cancer, and diabetes [1]. The COVID-19 pandemic highlighted the severe scarcity of healthcare resources which includes the number of physicians as well as access to screening tests and medical imaging [2]. While there is a rapidly increasing demand for medical imaging in the hospital, there is an alarming decline in the number of radiologists available to read the growing number of imaging studies. One previous study conservatively predicted that the demand by 2030 for radiologists would rise to a staggering 62,000 radiologists, yet there will only be a predicted 33,700 radiologists available [3,4]. Without drastic advancements in technology to assist in reducing the time spent reading each imaging study, radiologists will be unable to accommodate the increasing orders for imaging leading to further radiologist burnout [5]. This discrepancy in the number of radiologists can have severe ramifications in regards to patient care including missed or delayed diagnoses.

Previous county-level analysis of the US radiologist workforce utilized public datasets from 2014 and earlier which showed negative associations between county income and radiologist supply [6]. There are very few, recent studies that assess radiologists' geographical distribution despite an almost near-double increase in computed tomography and ultrasound tests ordered from 2010 to 2020 [7].

This surge is seen especially after the resolution of the COVID-19 pandemic and more specifically in underserved communities where an uptick in imaging studies like breast cancer screenings was observed. Essex County, New Jersey (NJ), is home to a large underserved community that observed a significant increase in the number of screening mammograms following the COVID-19 shutdown [8]. Without a sufficient supply of radiologists to read these screening mammograms in a timely manner, patient outcomes could be severely affected if a diagnosis were delayed or missed [9]. However, there are no studies that assess if the supply of radiologists in NJ in underserved areas such as Essex County is adequately sufficient to handle the increase in imaging volume. The primary objective of this study is to evaluate the geographical distribution of radiologists in relation to county income in NJ in hopes to identify any disparity in patient care in the field of radiology.

This article was previously presented in the form of an electronic poster presentation at the 2023 American College of Radiology Annual Meeting on May 6, 2023.

## Materials and methods

To ultimately evaluate disparities in radiologist densities between different counties, a list of currently active and practicing radiologists had to be generated. This study chose to utilize and query the NJ Health Care Profile to generate the aforementioned list. The database’s maintenance and oversight by the Division of Consumer Affairs made it a highly reliable option for the purposes of this study [10].

The advanced search option setting was used to generate alphabetized lists of actively practicing radiologists within NJ. Each radiologist was then searched online to determine all the locations, and ultimately the counties, in which they were practicing. Multiple locations within a single county were only listed as one county location due to the focus of the study pertaining to the actual counties themselves rather than the towns comprising the county. Any physician practicing outside of NJ was disregarded. Once a physician was found to be practicing in at least one location within the state of NJ, they were further stratified on the basis of being either a diagnostic radiologist or an interventional radiologist.

Using the US Census database, the median household income and population size for each county was recorded in the year 2021. All of the counties were then ordered by their median household so that the counties could be stratified into the following three different income categories: counties in the top one-third of median household income, counties in the middle one-third of median household income, and counties in the bottom one-third of median household income.

Using the substratifications by radiology specialty type, household income, population size, and counties, Microsoft Excel (Microsoft, Washington, USA) was used to carry out chi-squared analyses and descriptive statistics on these different subgroups, including many proportions and ratios using the stratified dataset. This allowed the study to use the ratio of the population size of each county to the number of radiologists in each county to serve as a surrogate marker for disparities following a standardization to account for differing population sizes between all of the different counties within the state. This ratio was subsequently used to compare disparities in the amount of practicing radiologists within a county on the basis of the income level within which the county fell into. A p-value less than 0.05 was considered statistically significant.

## Results

After querying the NJ Health Care Profile for all actively practicing radiologists within the state of New Jersey, the study identified 1,186 board-certified radiologists. Of these 1,186 radiologists, 86% (1,038 physicians) are solely diagnostic radiologists, while 14% (148 physicians) are interventional radiologists. As a result of many radiologists covering locations in multiple counties, the study found 1,555 county-based coverages.

Subsequent to stratifying counties by median household income in 2021, three groupings of the counties were created by splitting the counties into the following: in the top one-third of median household income, in the middle one-third of median household income, and in the bottom one-third of median household income. The study found that of the 1,555 county-based coverages, 44.37% (690 coverages) of them were in counties in the top one-third of median household income. About 25.15% (391 coverages) were in counties in the middle one-third of median household income, while 30.48% (474 coverages) were in counties in the bottom one-third of median household income (Table 1). A subsequent chi-squared analysis of the county-based coverages was performed resulting in a p-value of less than 0.001.

**Table 1:** County population sizes and corresponding radiologist counts in NJ

County sub-group	County	Median income	Population	# of radiologists	Individuals/radiologist
Top one-third of median household income	Morris	$123,727	491,845	71	6927
	Hunterdon	$123,373	124,371	26	4784
	Somerset	$121,695	328,934	51	6450
	Monmouth	$110,356	618,795	146	4238
	Bergen	$109,497	932,202	182	5122
	Sussex	$101,645	140,488	15	9366
	Middlesex	$96,883	825,062	199	4146
Middle one-third of median household income	Burlington	$95,935	445,349	49	9089
	Gloucester	$93,208	291,636	36	8101
	Union	$87,369	556,341	92	6047
	Mercer	$85,687	367,430	71	5175
	Warren	$85,163	105,267	20	5263
	Hudson	$79,795	672,391	62	10845
	Passaic	$78,386	501,826	61	8227
Bottom one-third of median household income	Ocean	$76,644	607,186	71	8552
	Cape May	$76,237	92,039	14	6574
	Camden	$75,485	506,471	130	3896
	Salem	$67,898	62,385	3	20975
	Essex	$67,826	798,975	155	5155
	Atlantic	$66,473	263,670	78	3380
	Cumberland	$58,397	149,527	23	6501

**Figure 1:**
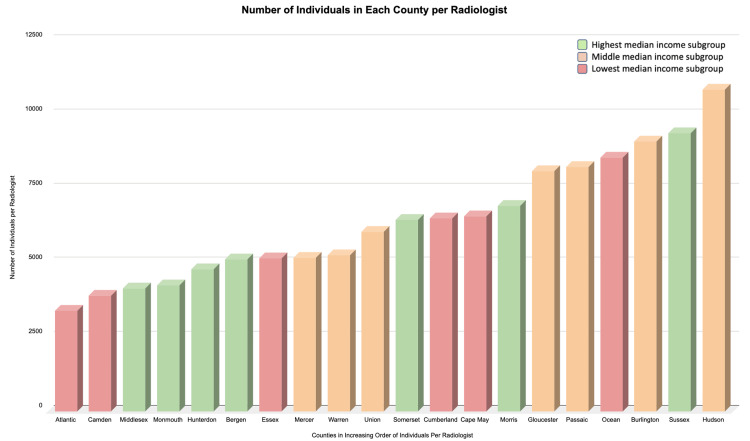
Graph demonstrating the number of individuals in each county per radiologist

The national percentage of individuals living below the poverty level in 2021 was estimated to be 12.8% [11]. Of the 21 counties that make up the state of NJ, five were found to have a higher percentage of individuals living below the poverty level compared to the national percentage of 12.8% (Figure 1). Hudson County was found to have the highest percentage of individuals living below the poverty level within the state with 16% living below the poverty level.

As aforementioned, to standardize the number of radiologists covering an area by the population size of a given county, ratios of individuals to radiologists were compared across the 21 counties to generate the number of patients per single radiologist (Table 2 and Figure 1). The average number of individuals per radiologist was 5,861 in the top one-third of the median household income county subgroup, 7,535 in the middle one-third of the median household income county subgroup, and 7,861 in the bottom one-third of the median household income county subgroup. A subsequent chi-squared analysis of the individuals per radiologist stratified by county income level was performed resulting in a p-value of less than 0.001.

**Table 2:** Statistical analysis of radiologist distribution by income bracket subgroup

Statistical comparison of radiologist coverage		
Chi-squared analysis of radiologist coverage by county income bracket	Highest one-third of income bracket: 690	p<0.001
	Middle one-third of income bracket: 391	
	Lowest one-third of income bracket: 474	
Chi-squared analysis of radiologist coverage by county income bracket	Highest one-third of income bracket: 5,861	p<0.001
	Middle one-third of income bracket: 7,535	
	Lowest one-third of income bracket: 7,861	
A p-value less than 0.05 is considered statistically significant		

## Discussion

There is a clear discrepancy between the need for radiologists and the availability of radiologists per given geographic area in the state of NJ. When assessing the geographic distribution of radiologists within the state, it becomes apparent that there is an enormous amount of variability in the number of people per available radiologist within certain towns and counties.

The present study found 1,555 county-based coverages by radiologists within the state, and of these, the largest percentage of coverages (44.7%) were in counties that fell into the top one-third of median household income relative to all counties within the state. Statistical analyses demonstrated a significant difference in county-based coverage by radiologists depending on county income stratification. Therefore, from a public health standpoint that underscores health equity, this is an extremely alarming trend especially given the already apparent disparity in the rates of certain radiologic procedures, such as diagnostic and screening mammograms, within low-income communities [12]. An inequitable distribution of radiologist coverage as evidenced by the present study can further serve to exacerbate these community-wide systemic health disparities [13].

The number of patients per radiologist within a certain categorical stratification was used as a surrogate measure for entities such as healthcare resource allocations and the burden on medical professionals, both of which are critical factors that ultimately determine the quality of care that a patient receives [14]. With decreased resources per patient and an increased burden on medical professionals, health outcomes are negatively affected. The present study calculated the ratios of individuals to available radiologists within different counties and towns to elucidate the current state of radiologists and the healthcare burden within the state. Statistical analyses demonstrated significant differences in patients per radiologist depending on county income level. Alarmingly, the bottom one-third of the median household income county subgroup had the largest patient-to-radiologist ratio (7,861 patients per radiologist compared to 5,861 patients per radiologist in the highest one-third of the income subgroup). This is yet again extremely concerning given that there is already an abundance of evidence to suggest that lower-income communities are in greater need of healthcare resource allocation [15].

Despite the importance and significant implications of the results of this study, it is also important to acknowledge a few limitations of the study. The data for the study regarding the populations of each county within the state of NJ was limited by the most recent US Census Bureau report which was in 2021. Therefore, any systemic changes that may have been instituted to remedy statewide health disparities after this year may not be accurately reflected. Additionally, querying the NJ Health Care Profile did not allow for the investigators of this study to account for teleradiology within counties. Despite these limitations, the results of this study demonstrated clear significance in the disparities pertaining to radiology within the state. Further longitudinal studies to assess radiologist recruitment efforts are needed to determine which counties are successfully recruiting more radiologists.

## Conclusions

Many public health efforts and initiatives to streamline equitable healthcare have displayed the need for greater medical resource allocation in low-income communities, particularly due to the greater need present within these communities. However, the current study shows that the field of radiology specifically within the state of NJ has yet to address this concern.

It is apparent that the highest income communities have less patient burden for radiologists, a surrogate for patient outcomes and health within the community as a whole. There needs to be a statewide re-evaluation of current policies and initiatives to redirect the geographic distribution of radiologists to better reflect the number of patients in a given geographic location rather than the current trend of following geographic income levels. NJ is particularly vulnerable to systemic community-based health disparities rooted in socioeconomic disparities due to having many counties (5 out of 21) that are even below the national poverty level.

As we continue to evaluate what can be done to improve community health both statewide and nationally, it is imperative that we explore the avenue of redirecting radiologists geographically to create uniformity in the number of patients per radiologist in a given geographic county and area.
